# Extracellular vesicles: key mediators in *in vitro* embryo production

**DOI:** 10.3389/fvets.2025.1641966

**Published:** 2025-08-20

**Authors:** Mostafa Pournourali, Nahid Mizban, Roxana Ehsani, Somayeh Ebrahimian, Touba Nadri, Nima Azari-Dolatabad

**Affiliations:** ^1^Reproductive Biotechnology Research Center, Avicenna Research Institute, ACECR, Tehran, Iran; ^2^Sina Fanavaran Mandegar Company, Alborz Science and Technology Park, Kamalshahr, Iran; ^3^Department of Molecular Biotechnology, National Institute of Genetic Engineering and Biotechnology (NIGEB), Tehran, Iran; ^4^Avicenna Fertility Centre, Avicenna Research Institute, ACECR, Tehran, Iran; ^5^Department of Animal Science, College of Agriculture, Urmia University, Urmia, Iran; ^6^Department of Internal Medicine, Reproduction and Population Medicine, Faculty of Veterinary Medicine, Ghent University, Merelbeke, Belgium; ^7^Department of Animal Science, University of Tennessee Institute of Agriculture and AgResearch, Knoxville, TN, United States

**Keywords:** extracellular vesicles, oocyte maturation, fertilization, embryo development, intercellular communication

## Abstract

Nano-sized extracellular vesicles (EVs) possess a lipid bilayer and are secreted from cells into their surrounding environment. The transport of multiple biomolecules, including DNA together with RNA, microRNAs (miRNAs), lipids, proteins, and metabolites, happens through biofluids via EVs for intercellular communication. Extracellular vesicles play crucial roles during the *in vitro* embryo production (IVEP) process. Specifically, the maturing oocyte benefits from EVs that facilitate cell-to-cell communication and transfer important biomolecules, which improve oocyte development potential. Moreover, EVs help establish important molecular control needed for oocytes to advance into the metaphase II phase, which enables proper fertilization events. In fact, the fertilization process depends heavily on EVs because seminal plasma-derived EVs play an essential role during fertilization, and they improve sperm motility as well as capacitation and the acrosome reaction, which are required for successful fertilization. EVs transport proteins together with RNAs, which enhance sperm capacity to fertilize. Embryos benefit from the optimal growth environment, which is maintained by oviduct and uterus-derived extracellular vesicles (EVs), as they support proper gene expression regulation. EVs produced in the oviduct enable embryo development, and those released by the uterus serve as communication channels for embryo-maternal environment integration required during implantation. These vesicles contain bioactive molecules such as miR-21, miR-26a, and HSP70, which are involved in key reproductive functions including granulosa cell (GC) signaling, oocyte maturation, and sperm function regulation. Overall, the reproductive system relies heavily on EVs because these vesicles manage oocyte development as well as the process of fertilization and embryonic development. The communication features of EVs using regulatory molecules indicate their potential role in assisted reproductive technologies (ARTs). Advancing our knowledge regarding EVs' mechanisms will support the development of novel strategies to enhance IVEP outcomes. This review provides an overview of the current understanding of the roles of EVs in oocyte maturation, fertilization, and embryo development.

## 1 Introduction

Population growth triggers the increasing worldwide need for animal-based food products, including dairy items and meat products (1–3). Consequently, the reproductive performance of farm animals has been improved through the widespread application of assisted reproductive technologies (ARTs), particularly *in vitro* embryo production (IVEP) (4). According to the International Embryo Technology Society (IETS), more than one million bovine embryos are produced *in vitro* annually worldwide, highlighting the scale and impact of these technologies (5). Over the past two decades, the animal breeding field has realized the usefulness of IVEP to enhance genetic advancement and reproductive efficiency (6). In this context, assisted reproductive techniques (ARTs) such as *in vitro* fertilization (IVF), intracytoplasmic sperm injection, somatic cell nuclear transfer, and *in vitro* maturation (IVM) are used for breeding cattle, buffalo, goats, as well as sheep and camels (7, 8). Similarly, IVF serves as an essential ART for human fertility, as it offers a solution for individuals affected by infertility (9, 10). Thus, IVF plays a dual role since it improves livestock breeding methodologies and facilitates human reproductive medicine (11).

**Figure 1:**
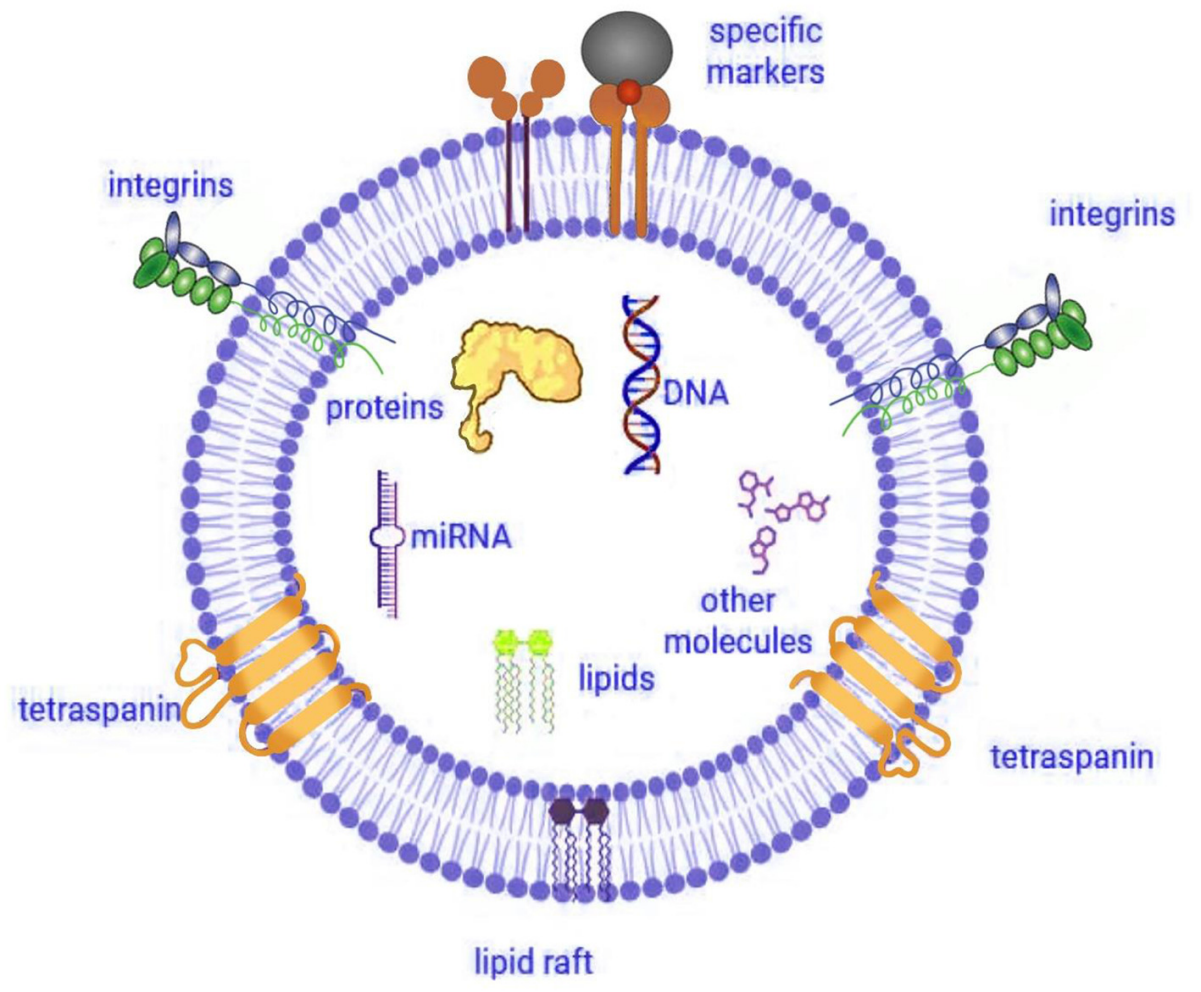
Structure and cargo of extracellular vesicle. Illustration created using Digital Paint and Adobe Photoshop 2023 and was inspired by Truby et al. (319).

Furthermore, researchers working in the field of IVEP have dedicated substantial efforts to developing improved strategies for assessing embryo quality under laboratory conditions (10, 12–14). Research studies have identified EVs as essential components responsible for cell-to-cell communications as well as embryonic developmental processes. EVs function primarily as carriers of bioactive molecules-such as proteins, mRNAs, and miRNAs-facilitating intercellular communication within reproductive tissues (Figure 1) (15, 16). Therefore, embryo development, together with implantation, depends on this vital molecular transfer mechanism that enables cell-to-cell communication (17, 18).

To explore these functions, researchers have applied EVs for IVEP investigation in cattle (19), horses (20), dogs (21), mice (22), pigs (23, 24), and humans (25). In addition, EVs in reproductive biology offer researchers critical cellular information and demonstrate potential usage as embryo viability predictors. Notably, functional characteristics of EVs mainly result from their protein cargo. These vesicles contain multiple tetraspanins, including CD9, CD63, and CD81, that assist cells with signaling activities and membrane processes (26). Moreover, the protein content of EVs includes essential proteins ALIX (27) as well as TSG101 (28) and MHC I (16) and HSP90 (29) together with integrins (α2, α6, β1, and β4) which facilitate critical embryo-maternal signaling processes (30).

This review discusses the essential functions of EVs throughout the IVEP process. Specifically, it begins with an explanation of EV types and their creation processes before discussing isolation and analytical methods. Finally, the review will investigate how these EVs affect IVF stages, starting from the expansion of cumulus cells up to embryo hatching, while demonstrating their ability to regulate embryonic developmental processes.

## 2 The classification of EVs

EVs are lipid bilayer-enclosed particles that are naturally released by most cell types into the extracellular space. They function as central mediators of intercellular communication by transporting a wide range of biomolecules, including RNAs, proteins, and lipids (31–33). According to MISEV2023 guidelines published by the International Society for Extracellular Vesicles (ISEV), EVs should be classified using operational terms based on measurable characteristics such as size, density, and biochemical composition. Commonly accepted categories include small EVs (sEVs; < 200 nm), medium/large EVs (m/lEVs; >200 nm), and apoptotic bodies (Table 1). The use of terms such as “exosomes” or “microvesicles” is not recommended unless their specific biogenesis pathways are experimentally confirmed (34).

**Table 1:** Classification of EVs.

**Types of extracellular vesicle**					
**No**	**Type**	**Size range**	**Origin**	**Markers**	**Functions**
1	Small EVs (sEVs) (320)	< 200 nm (321)	Endosomal (Multivesicular bodies) or undefined (322)	CD9, CD63, CD81, TSG101, Alix (323)	Involved in intercellular communication, cell motility, differentiation, proliferation, apoptosis, immunity (324, 325)
2	Medium/Large EVs (m/lEVs) (326)	>200 nm (16)	Plasma membrane budding (327)	Annexin A1, Integrins (328)	Participate in cell communication, tissue repair, coagulation, and immune responses (327)
3	Apoptitic bodies (329)	500–5,000 nm (330)	Formed during programmed cell death (apoptosis) (330)	Histones, fragmented DNA (331)	Facilitate clearance of apoptotic cells, may influence immune response (332)

This table presents the main categories of EVs based on MISEV2023 recommendations: small EVs (sEVs), medium/large EVs (m/lEVs), and apoptotic bodies. It outlines their approximate size ranges, modes of biogenesis, representative markers, and general features relevant to physiological and pathological functions.

Small EVs, typically ranging from 30 to 150 nanometers in diameter, are often associated with endosomal origin. They form as intraluminal vesicles within multivesicular bodies (MVBs), which then fuse with the plasma membrane to release their contents into the extracellular environment. Although commonly referred to as “exosomes” in earlier literature, MISEV2023 recommends using the term only when the endosomal origin has been experimentally verified (35–37).

Ectocytosis allows the release of m/lEVs from 50 to 1,000 nanometers in size through direct outward budding from plasma membranes. The formation process of plasma membrane-derived exosomes sets them apart from other exosomes that develop from endosomes (38, 39). The formation of m/lEVs proceeds differently from sEVs because it takes place from the plasma membrane surface instead of intracellular membranes or the endosomal system (40). On the other hand, apoptotic bodies take a wider size range from 1 to 5 μm in diameter during programmed cell death (41). These various types of EVs play significant roles in different biological processes, including cell motility (42–44), differentiation (45–47), proliferation (48), apoptosis (49), reprogramming (49–52), and immunity (53). The involvement of EVs in these processes highlights their potential for clinical applications (54).

Although the current ISEV guidelines recommend using the terms “exosomes,” “microvesicles,” and “apoptotic bodies” with caution-unless supported by highly specific isolation and characterization techniques-these categories are still widely used in the literature due to their distinct biogenetic origins. Exosomes (30–150 nm) are formed within multivesicular bodies (MVBs) and released when MVBs fuse with the plasma membrane. Microvesicles or ectosomes (100–1,000 nm) are generated through direct outward budding of the plasma membrane. Apoptotic bodies (500–2,000 nm), in contrast, are produced during programmed cell death and often contain fragmented nuclear material, organelles, and cytoplasmic content (55).

**Figure 2:**
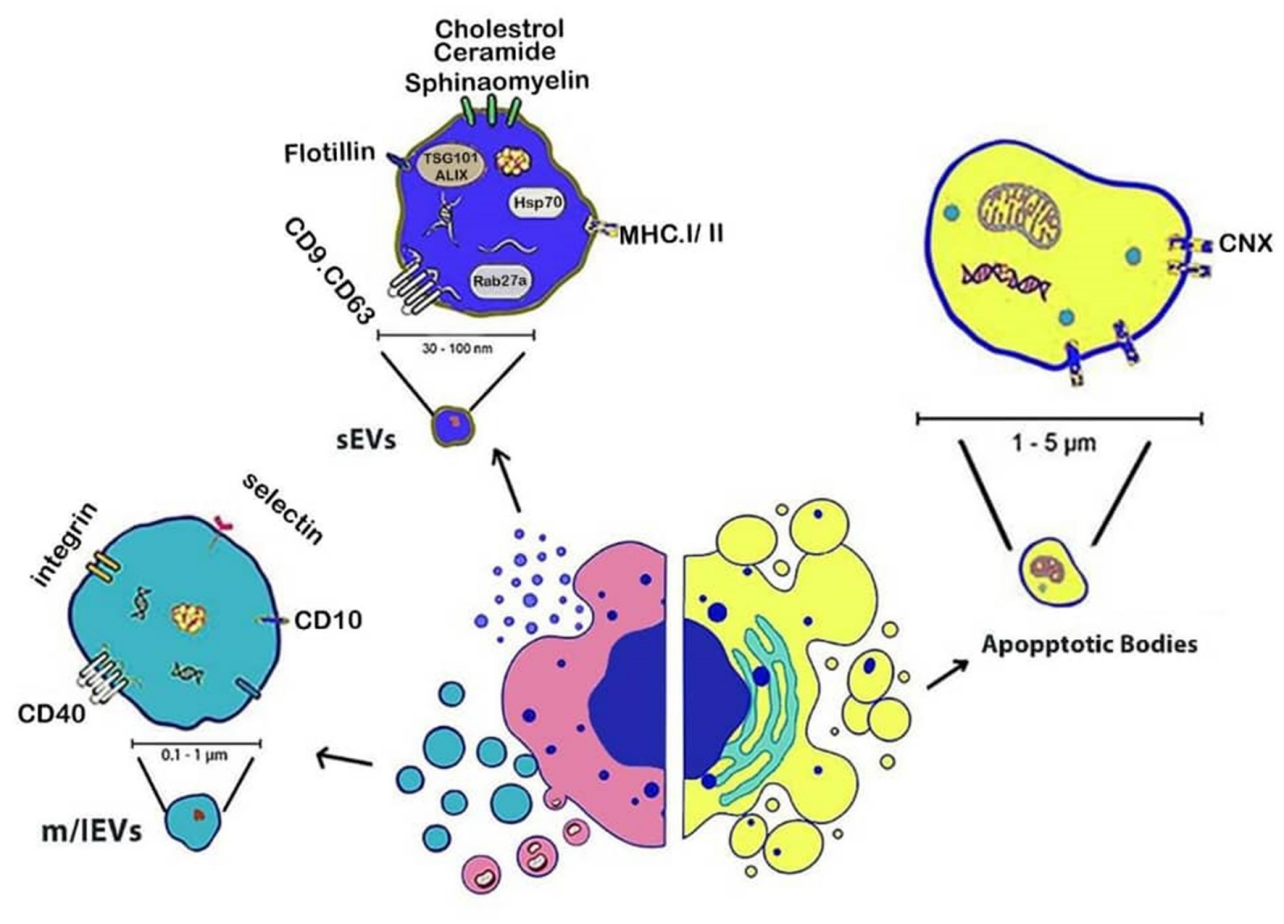
Schematic representation of EVs subtypes based on their biogenesis. small EVs (<200 nm) originate from the endosomal system, medium/large EVs (>200 nm) bud directly from the plasma membrane, and apoptotic bodies (>500 nm) are released during programmed cell death. Illustration created using Digital Paint and Adobe Photoshop 2023.

Intercellular communication through cellular exchanges regulates numerous physiological and pathological operations by sEVs and m/lEVs (56). Cargo of these EVs is transferred to nearby cells transfer to nearby cells (57, 58). The illustration in Figure 2 shows EVs are classified into sEVs, m/lEVs, and apoptotic bodies based on their size, biogenesis, and molecular characteristics, under MISEV2023 guidelines (Figure 2).

## 3 Cellular and biofluid sources of EVs in *in-vitro* embryo production

Extracellular vesicles originate from diverse cells and reproductive biofluids to function as vital items within natural communication paths between cells. During IVEP research, EVs from reproductive fluids and cells strengthen the maturation of oocytes and help with fertilization and contribute to embryo development (59, 60).

### 3.1 Reproductive fluids (follicular, oviductal, and uterine fluid)

Reproductive biofluids, including follicular fluid (FF), uterine fluid (UF), and oviductal fluid (OF), contain significant concentrations of EVs that enable essential communication processes between different reproductive cells. The FF-derived EVs employ different bioactive molecules that amplify oocyte competence as they accelerate both maturation stages of the cytoplasm and nucleus (61, 62).

The release of OF-derived EVs leads to sperm capacitation while promoting fertilization success by establishing maternal-embryo dialogue (63, 64). Many extracellular vesicles (EVs) present in follicular fluid are derived from granulosa and cumulus cells and contribute to oocyte development by transferring regulatory biomolecules. These EVs carry key microRNAs (miRNAs) such as *miR-21, miR-26a*, and *miR-375*, which modulate gene expression in follicular cells and enhance oocyte competence. Proteins such as *HSP70, Annexins*, and *OVGP1* have also been identified in follicular EVs and are linked to improved fertilization and embryo development outcomes. Studies have shown that the addition of follicular fluid-derived EVs to IVM media can improve oocyte maturation and subsequent embryo development (65, 66).

The delivery of EVs within UF plays an essential role in embryo development and creates endometrial receptivity, which results in better implantation rates in assisted reproduction procedures, as presented in (67).

### 3.2 Granulosa and cumulus cell-derived EVs

Oocytes receive regulation through EVs produced by granulosa cells (GCs) and cumulus cells that contain miRNAs, proteins, and growth factors, which influence both oocyte quality and embryonic development competency. These EVs are enriched with molecules that influence *mitochondrial function, epigenetic modifications*, and *cumulus expansion*, all of which are critical for proper oocyte maturation. For example, *miR-375* has been reported to improve mitochondrial membrane potential in oocytes, and *CD63* and *TSG101* have been used as markers for exosome-mediated signaling in cumulus–oocyte communication. Cumulus-derived EVs have also been associated with enhanced tolerance to *in vitro* stress conditions, contributing to better developmental potential post-fertilization (68). Multiple studies demonstrate that these EVs affect mitochondrial functions and epigenetic characteristics as well as *in vitro* production (IVP) embryo stress tolerance capabilities (69, 70).

### 3.3 Embryo-derived EVs

The early embryonic cells produce specialized signals that lead both to cell self-preservation and the regulation of neighboring cells during developmental processes. Recent studies have demonstrated that EVs function as essential communicative mechanisms that transport signals from cell to cell for vital embryo-maternal (71). It has been shown that the vital importance of embryo-derived EVs as cellular signaling mediators in both preimplantation embryonic development and the maternal environment is because their molecular components affect the embryonic development cycle and implantation, which leads to pregnancy establishment (72). A study showed that adding embryo-derived EVs from outgrowth embryos to culture medium enhanced both preimplantation embryonic development as well as implantation capability (73). The embryo-derived EVs contain gene-modifying molecules, which include mRNAs, miRNAs, and DNA fragments that reform gene expression and modify the cellular behavior of recipient cells. For example, blastocyst formation as well as implantation processes are influenced by specific EVs-delivered miRNAs (72). Embryo-derived EVs have been shown to carry common exosomal markers such as CD9, CD63, CD81, ALIX, TSG101, and HSP70, which contribute to embryo-maternal communication (74).

Also, new evidence shows that the embryo starts secreting EVs during early pregnancy, coming from the trophectoderm and inner cell mass. Likewise, the blastocoel fluid of preimplantation human embryos contains exosomes, which represent a specific subtype of EVs according to research (75). In addition, research suggests that human embryo-derived EVs have a connection to embryonic quality and can control maternal pregnancy recognition through modifications of endometrial epithelial cell transcript expression. Additionally, specific microRNAs, including miR-30c and miR-378, have been implicated in promoting trophoblast adhesion and regulating gene expression during implantation. Furthermore, research shows that maternal responses occurred only upon exposure to EVs derived from high-quality embryos, yet no detectable reactions were observed from EVs produced by degenerated embryos (76–78).

Embryo origin (*in vivo* or *in vitro*) together with culture conditions affect both embryonic EVs and maternal EVs concentration, size, and molecular profile, enabling changes in embryo-maternal signaling (71, 79, 80). Current research shows that EVs released into culture medium possess the potential to act as biomarkers that help evaluate embryonic quality and developmental competence. Embryos with poor developmental quality tend to produce increased concentrations of EVs, likely due to stress (81, 82).

Moreover, research indicates that EVs' dimensions present in human embryo culture medium serve as a non-invasive indicator for developmental competency in embryos (83). Research findings present inconsistent outcomes about the relation of EV size to embryo viability because some evidence links bigger EVs with healthy embryos, yet other studies show non-viable embryos generate larger quantities of large EVs that indicate stress symptoms. Research on bovine embryos produces contradictory results regarding the link between embryonic vesicle size and embryonic quality assessment (84, 85).

Further research reveals that human embryos release higher concentrations of EVs during later developmental stages, potentially reflecting increased cellular activity and structural complexity associated with processes such as blastulation (72, 86).

## 4 Methodological overview

Comprehensive information about the physicochemical properties of EVs-including size, shape, density, surface charge, and porosity, is essential for understanding their biological functions and interactions (87, 88). Several widely used characterization techniques assist in this analysis, including.

### 4.1 Nanoparticle tracking analysis (NTA)

Nanoparticle tracking analysis (NTA) is a widely used technique for the biophysical characterization of EVs. It operates based on the principle of light scattering and enables the tracking of the Brownian motion of individual nanoparticles in a liquid suspension (89). In the context of EV analysis, NTA tracks the movement of each particle through image analysis, measuring their velocity (90), which is correlated with their size (91). This technique is capable of examining the concentration and size distribution of EVs within a size range of ~50–1,000 nm (92). Moreover, NTA can also analyze the zeta potential, which reflects the surface charge of EVs. This measurement provides valuable information about vesicle stability, aggregation tendency, and interactions with biological membranes. NTA requires minimal sample preparation, and the analysis process is relatively quick (93, 94). Additionally, NTA offers a fluorescence mode that allows for the probing of EV surface antigens using labeled antibodies (95). The success of NTA heavily depends on proper sample preparation and the correct dilution factor (96).

### 4.2 Dynamic light scattering (DLS)

Dynamic light scattering (DLS) represents a widespread analytical method for nanoparticle size distribution measurements, with EVs represented as part of this group of nanoparticles (97). DLS measures scattering light intensity fluctuations produced by particles performing Brownian motion in suspension in order to calculate their hydrodynamic diameter. However, it is important to note that DLS does not provide information on particle concentration, which limits its utility when quantitative analysis of EVs is required (93, 98).

There are some advantages of using DLS including: (1) non-invasive and rapid: DLS is a non-destructive method that requires relatively small sample volumes, making it suitable for analyzing precious biological samples such as EVs (99). (2) Broad size range: the technique is capable of measuring particles ranging from ~1 nm to several micrometers, encompassing the typical size range of EVs (93). (3) High throughput: DLS can quickly provide size distribution data, facilitating rapid screening and analysis of multiple samples (100). The analysis of vesicular structures through DLS shows two major limitations due to its excessive detection of bigger particles and its incapability to locate small vesicles among larger ones. The technique fails to accurately measure diverse particle combinations found in heterogeneous mixtures because it cannot account for non-spherical vesicle shapes (101). Additionally it does not provide data regarding biochemical characteristics, origin or functional properties of EVs (93). Despite its limitations, DLS has been effectively employed in various studies involving EVs: (1) size distribution analysis: DLS has been used to assess the size distribution of EVs derived from different cell types, such as red blood cells and ovarian cancer cells, aiding in understanding their biophysical properties (93). (2) Quality control: the technique serves as a quality control measure to detect aggregation or changes in EV size distribution during isolation and storage processes (99). (3) Comparative studies: DLS has been utilized alongside other characterization methods, such as NTA and electron microscopy, to provide complementary data on EV populations (92, 101).

The detection of smaller particles remains challenging for this technique because larger particles within a mixture can prevent the successful detection of the smaller ones (101, 102). DLS demonstrates remarkable capability for measuring vesicles of various origins, such as red blood cells and ovarian cancer cells, regardless of its size measurement limitation (103). The diameter measurements from DLS are straightforward, but this technique lacks the capability to identify the origin or constituents of EVs (104). EVs' size analysis is possible with this tool yet researchers need supplementary equipment to uncover EVs' complete biological properties fully (105, 106).

Dynamic light scattering functions as a significant analytical technique for studying EVs by enabling fast and non-destructive distribution measurements of their sizes (107). The assessment of EVs demands awareness of DLS's two main constraints, which include the size measurement effect on bigger particles and insufficient biochemical data (108). Getting a full understanding of EVs requires the use of DLS alongside other analytical methods which reveal their molecular structure along with functional characteristics.

### 4.3 Tunable resistive pulse sensing (TRPS)

TRPS stands as a significant technique for determining both the size dimensions and concentration values of EVs (109). This technology allows researchers to perform unclouded analysis of individual sample constituents along with precise characterization of their profiles. TRPS provides effective characterization results for colloidal particles along with diverse nanoparticles and biomolecules in suspension from 50 nm particles through cellular dimensions, thus enabling investigations of cellular functionality and EV uptake (109, 110). TRPS encounters technical limitations concerning system stability, together with sensitivity issues. The stability of the system will decrease when particles block the pores. The usage of coating solutions that decrease surface binding washes of non-specific molecules has demonstrated improvement in measurement accuracy according to (111). Small particles present measurement difficulties because they become challenging to detect among background signals. The intensity of light scattering occurring in DLS techniques causes larger particles to overpower smaller ones when present together. System performance can be improved when technicians optimize three key components, such as noise reduction measures and sensitivity cutoff boundaries, with precise measurement performance protocols. The technical roadblocks do not limit TRPS's effectiveness as it continues to function as a flexible and highly effective analytical approach. The technique has found applications, including studying the DNA binding process to magnetic nanoparticles along with characterizing leukemia-derived EVs between 200 and 300 nm during their interaction with the extracellular matrix (ECM) (112). Research has extensively focused on TRPS to determine the size distribution of EVs in various studies (113, 114). TRPS exists in two specialized versions that function as delivery platforms for enzymes against Alzheimer's disease while simultaneously delivering anticancer miRNAs to tumor cells (115–117).

### 4.4 Flow cytometry

Flow cytometry operates as a strong analytical tool to assess EVs, including exosomes, through detecting laser-exited light scattering and fluorescent emissions from liquid streams containing these particles (118). Due to the small size of EVs, flow cytometry typically requires specialized approaches such as nano-flow cytometry (nFCM) or technical modifications to conventional instruments to achieve accurate detection. Flow cytometry accomplishes comprehensive structural analysis of EVs while measuring essential morphological and parametric characteristics of thousands of particles each second (119, 120). Flow cytometry provides a specific advantage as it enables precise counting and separation, and purification of EV populations present in suspension (121).

Unlike ultracentrifugation, which is a method for EV isolation, flow cytometry is an analytical technique that additionally enables EV sorting. This feature provides a key advantage, particularly when studying EV subpopulations (111). Analysis of EVs becomes possible through this method without needing previous isolation or concentration procedures when working with limited sample volumes or requiring speedy assessment processes (122).

Standard flow cytometry equipment proves inadequate for examining exosomes between 30 and 150 nm since their dimensions approach instrument detection levels and particle light scattering matches background noise. The development of advanced flow cytometers has included features for enhanced sensitiveness in addition to improved forward scatter detection and fluorescence amplification and high-resolution imaging functions (123, 124). The innovations enhance EV sizing resolution especially for small vesicles < 200 nm diameter (71), through fast classification and antigen measurement at the single vesicle level (125).

To perform flow cytometry analysis experts must be available together with state-of-the-art laboratories. The process of precise detection of EVs usually demands labeling with fluorescent dyes or antibodies yet these requirements might sometimes affect subsequent examination procedures. Measuring and identifying EV signals through flow cytometry becomes difficult due to the inherent limitations posed by their small dimensions and low refractive properties (126, 127). The advancement of both instrumentation techniques and labeling methods intends to resolve these analytical difficulties, thereby boosting the sensitivity and specificity of EV flow cytometric analysis. While flow cytometry allows high-throughput multiparametric analysis and sorting of EVs, it still faces limitations in sensitivity and resolution when analyzing small vesicles, unless advanced setups like nFCM are used.

### 4.5 Transmission electron microscopy technique

Transmission electron microscopy (TEM) operates as the fundamental approach to inspect biological components, specifically EVs, through evaluating their dimensions and morphological elements, and physical features (128). With electrons instead of light as the imaging source, TEM resolves down to the nanometer range, thus allowing scientists to study EV morphology in detail (129). The exposure of a thin sample layer to an electron beam produces an image that shows a diffraction pattern through electromagnetic lens detection of electron scattering. The technique enables both optic diameter measurements of the vesicles and evaluations of their structural condition (130, 131).

Biological specimens demand special laboratory handling before TEM analysis to keep their biological structures intact. TEM imaging of EVs exhibits multiple morphological patterns that include round and cup-shaped structures which represent their naturally diverse biological sources and operational tasks (132, 133). The TEM electron beam possesses the ability to create damage to biological specimens which creates distorted results that hinder correct interpretation (134, 135). Cryo-TEM serves as an investigative method to reduce both beam-induced damage and dehydration artifacts in studies of EVs (136). Samples undergo rapid vitrification under this method which safeguards their biological composition through the maintenance of vitreous ice instead of require fixation or dehydration applications. The ultrastructural integrity of EVs remains intact through Cryo-TEM analysis because it stops both the modification of structure and the relocation of elements (137).

Cryo-TEM represents an ideal approach to study biological molecules by avoiding deformations caused by dehydration while providing clear visualization of EVs alongside their membranous components and lumens (87). The study of EV biological functions requires accurate detection of specific proteins positioned inside their cargo (31, 138). The bright fluorescent signals from labeled proteins make it hard for TEM to view the labeled EVs. Researchers typically use immunogold labeling TEM to observe EVs because this method shows antibody-probed EVs under the microscope (139, 140). The localization technique depends on gold nanoparticles, which link to specific target protein antibodies to identify particular proteins both inside and outside EV structures under TEM analysis (131, 141).

The detailed characterization of EVs strongly relies on TEM together with its variant methods, including Cryo-TEM and immunogold labeling. These investigation methods reveal important information about EV shape and chemical structure, as well as working mechanisms that advance scientific knowledge of biological processes involving EVs.

### 4.6 Atomic force microscopy (AFM) for EVs characterization

Numerous studies have demonstrated the effective use of AFM for studying the physicochemical characteristics of EVs obtained from different biofluids such as blood, saliva, and synovial fluid, according to (142). High-resolution topographical imaging of EVs becomes possible through AFM because the technology works under near-physiological conditions while providing vital information about EV morphology and biomechanical properties, along with composition details (143, 144), significantly enhancing our understanding of these vesicles at both the single-vesicle and sub-vesicular levels (145).

The probing-tip interference with EV surfaces allows AFM to generate 3D topographic images through atomic-scale detection of mechanical surface interactions (146). The method delivers multiple advantages compared to electron diffraction-based techniques since it offers better sample management and enables damage-free imaging functions. The analysis of EVs by AFM works without fixing EVs, so scientists can maintain their natural state while preserving their structural integrity (147). AFM technology provides high-resolution observation of single and sub-vesicular EV structures, which helps scientists measure their dimensions and surface features (148). The biomechanical properties of EVs become measurable through AFM since it assesses both the elasticity and stiffness as well as adhesion characteristics of these vesicles, which assists researchers in understanding their cellular uptake capability and signaling mechanisms. AFM analysis requires minimal sample preparation because researchers avoid using damaging procedures, which avoids artifact formation during the observation of EVs (147, 149).

Technical issues hinder the application of AFM for EVs characterization, even though it offers various benefits. Experimental control under standardization remains essential because the natural state of samples from EVs changes considerably (150). Controls must be taken to address three sources of artifacts following AFM imaging because the technique remains highly sensitive to sample preparation, along with substrate interactions and scanning speed, leading to EVs' structural topographical distortions and mechanical deformations (151, 152). Low-throughput operations are a characteristic of AFM since it demands time-intensive expertise and analysis procedures (149).

When studying EVs characterization through mechanical assessment and topographic analysis of nanoscale features both AFM reveals itself as a powerful non-specific technology for sample examination without affecting underlying structures. To achieve valid experimental results as well as reproducibility researchers must optimize their experimental settings and standardize research methods in EVs identification work. The utility of automated AFM systems combined with machine learning-driven image analysis can advance to increase high-throughput characterization of EVs in the future.

To aid researchers in selecting the most suitable EV analysis techniques, Table 2 summarizes the parameters evaluated by each method, their primary advantages and limitations, representative instruments, estimated costs, and relevant publications.

**Table 2:** Summary of key analytical techniques for EV characterization.

**No**	**Technique**	**Parameters measured**	**Main advantages**	**Main limitations**	**Representative instruments**	**Approx. cost (USD)**	**Example studies**
1	NTA	Size, concentration, zeta potential, fluorescence	Real-time tracking; fluorescence mode; relatively easy setup	Limited resolution < 50 nm; dilution sensitive	NanoSight NS300 (Malvern), ZetaView (Particle Metrix)	$70,000–150,000	(333, 334)
2	DLS	Size distribution (hydrodynamic diameter)	Fast; simple; non-destructive	Cannot determine concentration; biased toward larger particles	Zetasizer Nano (Malvern)	$60,000–120,000	(335–337)
3	TRPS	Size, concentration, particle-by-particle analysis	High resolution; direct quantification; single-particle accuracy	Pore clogging; sensitive to sample viscosity	qNano Gold (IZON Science)	$50,000–90,000	(248, 338)
4	Flow Cytometry (nFCM)	Surface markers, size, fluorescence, sorting	High-throughput; specific surface marker analysis	Requires advanced setup (nFCM); resolution issues < 150 nm with standard cytometers	NanoFCM (NanoFCM Inc.), CytoFLEX S (Beckman Coulter)	$150,000–300,000	(131, 339, 340)
5	TEM/Cryo-TEM	Morphology, structure, vesicle integrity	High-resolution imaging; structural detail	Requires extensive sample prep; not quantitative	JEOL 1200EX, FEI Tecnai	$200,000–600,000	(87, 131, 340)
6	AFM	Topography, elasticity, mechanical properties	Imaging under near-native conditions; label-free	Low throughput; sensitive to sample prep artifacts	Bruker Dimension FastScan, JPK NanoWizard	$100,000–250,000	(150, 341, 342)

## 5 EVs in reproductive fluids and their role in *in-vitro* embryo production

**Figure 3:**
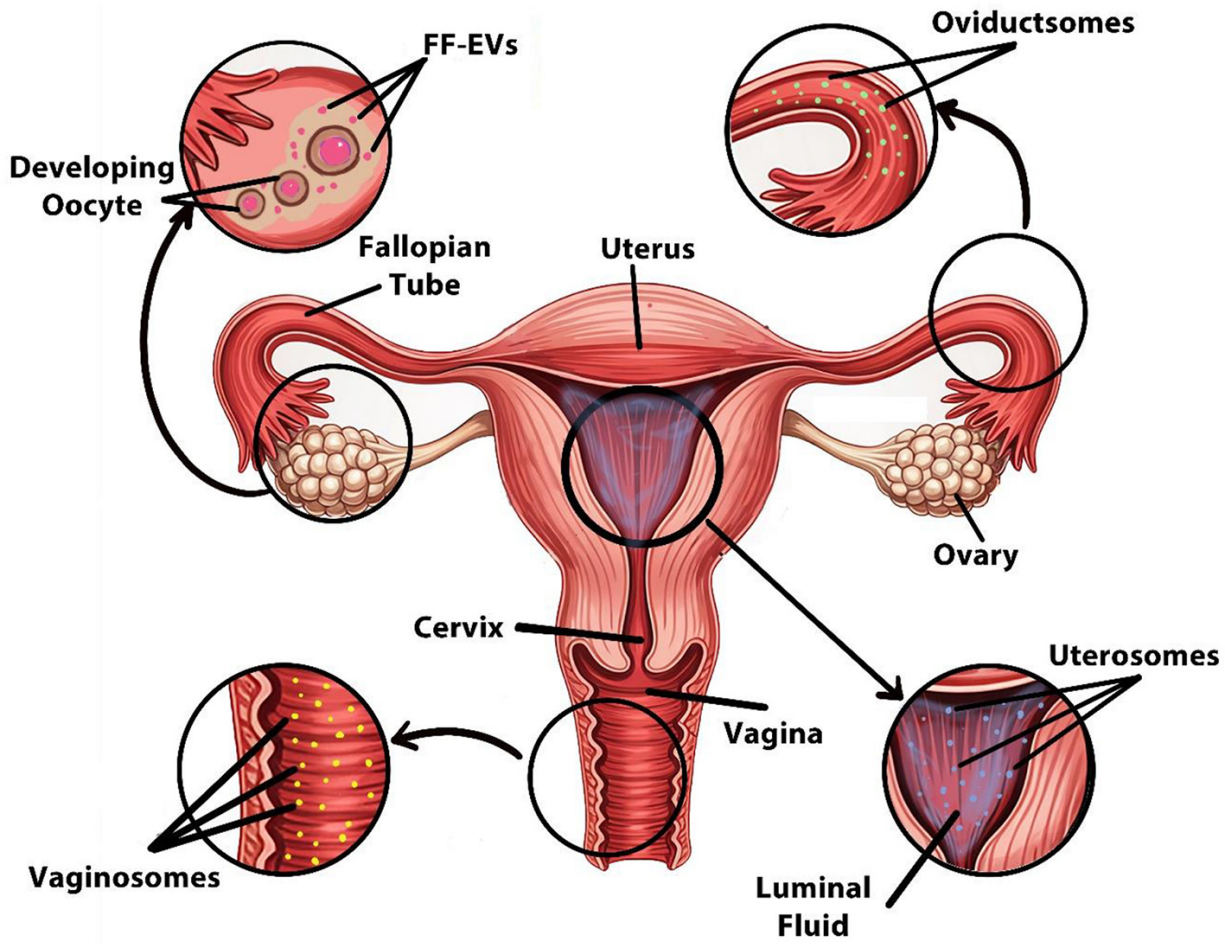
Sources of EVs in the female reproductive system. Designed using Digital Paint and Adobe Photoshop 2023.

Reproductive fluids such as FF, OF, endometrial fluid, amniotic fluid, and seminal fluid have been reported to contain EVs (Figure 3), which have been molecularly characterized by the presence of tetraspanins such as CD63 and CD81, and proteins like TSG101 and HSP70 (153). These EVs participate (e.g., miR-21, miR-132, miR-145) in oocyte maturation alongside fertilization and early embryonic development and implantation processes, making them critical mediators for IVP and ARTs (154).

Specifically, FF-EVs carry miRNA particles together with proteins and lipids, which are involved in granulosa cell signaling and oocyte maturation, thereby improving embryo development (155).

Similarly, oviductal EVs, often referred to as oviductosomes, contain specific glycoproteins, including OVGP1, Annexins, and HSP70, that serve to remodel the zona pellucida while improving sperm-oocyte binding performance. These EVs facilitate fertilization and early zygote development by transporting essential regulatory molecules. The hormonal control mechanism regulates secretion levels, which reach their maximum during the peri-ovulatory phase to enhance fertilization conditions inside the oviduct (156–158).

In addition, the embryo-maternal communication process relies on endometrial fluid–derived EVs that enhance endometrial receptivity by delivering miRNAs (such as miR-30d and miR-200c) and adhesion-related molecules (e.g., integrins αVβ3) to help activate crucial signaling pathways of early trophoblasts necessary for proper (159–161).

Moreover, the significant function of seminal EVs impacts male fertility, together with embryo quality performance. Seminal EVs influence sperm functionality and support capacitation, especially through delivery of prostasomes enriched with CD9 and enzymes such as P34H that modulate acrosome reaction and motility (162).

EVs found in amniotic fluid enriched in surfactant proteins and inflammatory mediators (e.g., IL-6, TNF-α), likewise, provide insights into maternal–fetal communication while scientists evaluate their potential as diagnostic markers for IVP embryonic health assessment (163).

Additionally, antimicrobial peptides together with defensins located in vaginal epithelial EVs known as vaginosomes help sustain proper vaginal microbiota equilibrium. The vesicles play a regulatory role in sperm selection along with early sperm survival based on hormone-controlled estrous cycle fluctuations (68, 164).

Finally, research has investigated EVs extracted from reproductive fluids because they may serve as embryonic biomarkers in ART and effective modulators to improve IVP success (165, 166).

## 6 Role of male reproductive tract EVs in *in vitro* embryo production

**Figure 4:**
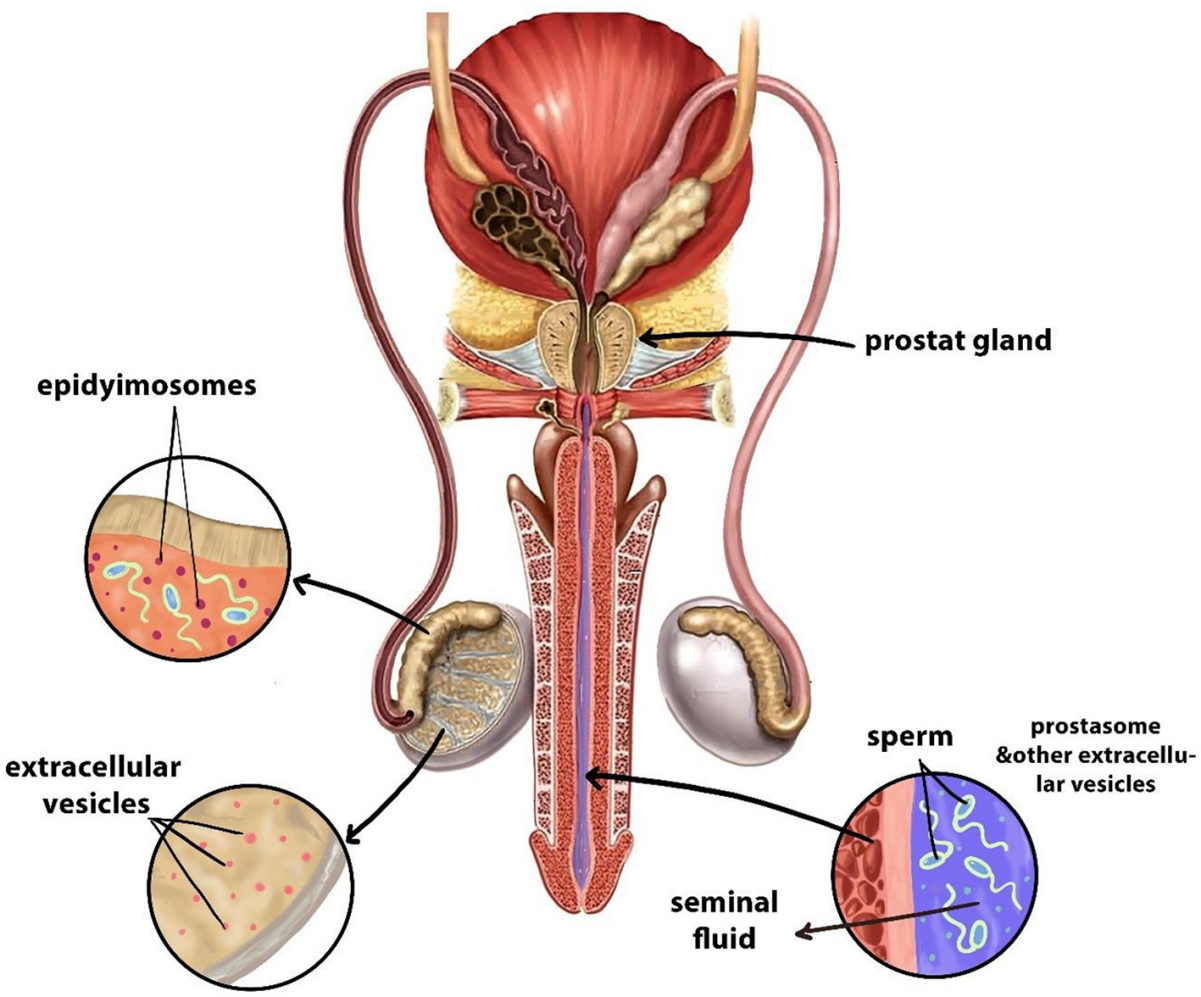
Sources of EVs in the male reproductive system. Designed using Digital Paint and Adobe Photoshop 2023.

Extracellular vesicles secreted by the male reproductive (Figure 4) tract assist the maturation of sperm cells while improving their capability to capacitate and fertilize, which enables successful IVEP. Notably, research has mainly focused on epididymal and seminal fluid-derived EVs because they impact sperm physiological processes. Also, the testis together with the epididymis, vas deferens, and prostate actively secrete EVs (167–172).

Specifically, EVs released by the epididymis, epididymosomes, contain proteins like CRISP1 and miRNAs such as miR-888 and miR-891a help guide essential processes of sperm development, which are known scientifically as epididymosomal activities. These vesicles carry bioactive molecules to sperm cells to promote membrane changes and increase mobility and better fertilization ability (171, 172). Interestingly, the biochemical structure of epididymosomes changes throughout the epididymal regions, which leads to distinct effects on sperm development stages. The different compositions of vesicles among epididymal regions potentially make them suitable to serve as biomarkers for sperm selection during IVF procedures (173).

In addition, the male reproductive tract cells originating from different areas produce a variety of EVs that are now used to be classified as prostasomes (173). The essential functions of these EVs include protecting spermatozoa from oxidative stress, as well as regulating immune responses in the female reproductive tract following fertilization and sperm-oocyte interaction facilitation (174, 175). They are enriched with bioactive molecules such as tetraspanins (CD9, CD63, and CD81), enzymes like P34H and HSP70, and regulatory RNAs including miR-34c and miR-210, which play key roles in enhancing sperm motility, acrosome reaction, and fertilization efficiency (176–178).

Therefore, the male reproductive tract EVs both maintain fertility functions along with opening new opportunities for ARTs. These EVs demonstrate potential applications as supplementary agents in IVP procedures that enhance sperm capacitation capabilities, the handling process, and subsequent fertilization results. They are enriched with bioactive molecules such as tetraspanins (CD9, CD63, and CD81), enzymes like P34H and HSP70, and regulatory RNAs including miR-34c and miR-210, which play key roles in enhancing sperm motility, acrosome reaction, and fertilization efficiency. Furthermore, further study of these vesicles as well as their active substances could develop new approaches to assess sperm quality together with optimizing cell culture environments within IVP systems (174, 175).

## 7 Role of female reproductive tract EVs in *in vitro* embryo production

EVs originate from ovarian follicular cells (Figure 3) as well as oviductal epithelial cells and *in vitro*-fertilized embryos, and endometrial cells to mediate crucial biological functions during oocyte maturation, fertilization, and embryo-maternal interactions. Recent studies using human, bovine, and equine models reveal that EVs hold significant promise for increasing the effectiveness of IVP (179, 180).

### 7.1 Follicular fluid EVs and oocyte maturation

Interestingly, FF itself and FF-EVs are a rich supply of bioactive molecules essential for controlling cumulus cell activity and oocyte maturation. According to recent research, supplementation of FF during *in vitro* maturation can enhance cumulus growth and improve the quality of the resulting embryo in a dose-dependent manner (181). Specifically, the microenvironment of the oocyte contains FF that releases EVs by the trio of granulosa cells, cumulus cells, and theca cells (182). The bioactive contents owned by EF-derived EVs regulate oocyte growth and maturation through the delivery mechanisms of miRNAs, proteins, and lipids. Notably, several specific molecules such as miR-130b, miR-21, BMP15, and GDF9 have been identified in FF-EVs, contributing to cumulus cell expansion, inhibition of apoptosis, and promotion of oocyte meiotic competence (65). In addition, several studies have demonstrated that supplementation of FF-EVs in IVP culture media enhanced developmental competence, together with higher blastocyst formation rates (16, 183–185).

### 7.2 Oviductal EVs and early embryo development

The oviduct serves as an essential environment where fertilization occurs, along with early embryo maturation taking place. The OF released by epithelial cells generates EVs known as OF-EVs, which engage in continuous interactions with gametes and early developing embryos. Research shows that these vesicles improve sperm capacitation while increasing both sperm viability and fertilization potential because of carrying proteins and regulatory RNAs to target cells (186). These OF-EVs have been shown to contain OVGP1, annexins, and small RNAs like miR-375, which facilitate sperm-egg interaction, enhance embryo cleavage, and improve zona pellucida remodeling (187). Moreover, it has been revealed that supplementation of OF-EVs into culture media enhanced both cleavage rates and blastocyst formation, thus confirming their role in improving IVP systems (188). Consequently, studies indicate that OF-EVs as biological substances that can enhance IVP outcomes (162).

### 7.3 Endometrial EVs and embryo-maternal communication

The maternal communication pathway between embryo and tissue becomes active during the post-fertilization period due to EM-EVs' role as essential mediators. These vesicles, secreted by endometrial epithelial and stromal cells, carry signaling molecules that aid trophoblast adhesion during embryo implantation (189). Recent findings highlight the presence of miR-30d, integrin αvβ3, LIF, and HSP70 in EM-EVs, which modulate immune tolerance and promote trophoblast attachment and invasion into the maternal endometrium (190–194).

Furthermore, the process of successful implantation needs maternal immune tolerance to maintain pregnancy because EM-EV signaling helps regulate this essential aspect (195). Importantly, the enhanced maternal endometrium responsiveness enables EM-EVs to prove useful for embryo transfer processes in reproductive medicine assistance (195). As a result, research now examines EM-EVs for use as implantation success biomarkers and treatment options to enhance the implantation potential of IVP-derived embryos, since implantation stands as a primary challenge for ART success rates.

## 8 The role of seminal plasma and oviductal EVs in IVP

Spermatogenesis depends on EVs within seminal plasma and the female reproductive tract because these vesicles regulate sperm function during capacitation as well as fertilization processes (196, 197). Notably, these EVs maintain their impact on sperm performance from before to after fertilization and create new possibilities for improving the techniques of sperm preparation, cryopreservation, and sperm-oocyte interaction in IVP.

### 8.1 Seminal plasma EVs and their impact on sperm function

Seminal plasma consists of various testicular and epididymal fluids alongside fluids from accessory sex glands that keep spermatozoa enveloped from ejaculation time until after ejaculation occurs (198). Importantly, EVs isolated from seminal plasma transport biological molecules that direct sperm maturation and fertilization processes (199). Given that sperm cells are largely transcriptionally inactive, the relevance of this EV-mediated regulation lies primarily in post-transcriptional mechanisms and direct cellular interactions (200). Seminal plasma EVs, including exosomes, interact with spermatozoa and other cells in both the male and female reproductive tracts (201). This interaction involves transferring regulatory cargo such as miRNAs, proteins, lipids, and various small non-coding RNAs (sncRNAs) like tRNA, Y RNA, piwi-RNA, and ribosomal RNA, which are present at high concentrations in seminal plasma EVs. While mRNA and DNA have also been found within EVs, their specific roles in intercellular communication and gene expression within the recipient sperm remain under investigation (193, 202). The pro-survival effect of these vesicles on sperm cells stems from the ability of their miRNAs to prevent apoptotic gene expression, including BAX and CASP9, and CASP3 genes (203). This protective effect also helps protect sperm against oxidative stress, which is essential for maintaining sperm quality and function (204, 205).

Furthermore, researchers have shown that low-fertility sperm function improves when incubated with high-fertility donor EVs extracted from seminal plasma during *in vitro* production in both bovine and equine species (206). Also, the research demonstrates how seminal plasma-derived EVs could serve as valuable tools for improving sperm selection techniques and generating better results in IVF applications (199). Also, the research demonstrates how seminal plasma-derived EVs could serve as valuable tools for improving sperm selection techniques and generating better results in IVF applications (171). These improvements are mediated by the molecular cargo of EVs, which includes proteins such as PDIA4, Gelsolin, and CRISP1—known to enhance sperm capacitation, motility, and acrosome reaction—while proteins like SNF8, aldehyde oxidase, and Mucin 15 have been linked to poor semen quality. Additionally, seminal EVs carry miRNAs (e.g., miR-21-5p, miR-222) and other small non-coding RNAs (tRNA, piRNA, and Y RNA) that post-transcriptionally regulate sperm function, despite the limited transcriptional activity of mature sperm. These molecules influence sperm performance by modulating calcium signaling (e.g., via CatSper channels), membrane fluidity, and apoptotic pathways, as well as protecting against oxidative stress. Moreover, seminal EVs interact with the female reproductive tract by promoting uterine immune tolerance and decidualization, thereby enhancing the environment for fertilization and implantation (207, 208, 208–213).

### 8.2 Application of EVs in sperm cryopreservation for IVP

Cryopreservation is commonly applied in ART; however, it causes such severe damage to sperm that it negatively affects their ability to move and remain viable, together with their fertility potential. Recent studies demonstrate that the addition of EVs from seminal plasma combined with the oviduct can minimize the negative impacts caused by freezing on sperm cells. The addition of EVs to freezing procedures demonstrates their ability to protect sperm membranes while boosting post-freeze motility, which results in better blastocyst formation (199). This protective effect is thought to be mediated by EVs carrying heat shock proteins (e.g., HSP70), aquaporins, and annexins, which help stabilize the sperm plasma membrane and reduce cryo-induced damage (207, 214–216).

Interestingly, the temporary reduction of sperm mobility occurred when sperm interacted with porcine oviductal EVs, yet their survival rate improved (44). However, successful IVP requires proper optimization of EV concentration because such optimization remains essential for clinical implementation. Moreover, EVs from the oviduct and seminal plasma also deliver miRNAs (such as miR-34c and miR-19b) that regulate oxidative stress responses and support mitochondrial function in sperm cells during cryopreservation (68).

### 8.3 Oviductal EVs and their role in sperm capacitation for IVP

The absence of complete oviductal secretions during *in vitro* sperm capacitation prevents a proper execution of sperm storage selection and activation processes (217). Recent discoveries show OF-EVs fulfill three essential roles by enhancing zona pellucida sperm binding (218) and enforcing sperm hyperactivation alongside capacitation changes (219), along with controlling sperm survival inside the oviduct before ovulation (220). These effects are attributed to the transfer of specific proteins such as OVGP1, PMCA4, and CatSper regulators, which induce calcium signaling and promote tyrosine phosphorylation—key steps in capacitation (221–223).

In equine species, the addition of EVs isolated from equine oviduct tissue during IVF increases the success rates because they replicate natural fertilization signals (224). Similarly, adding oviductal EVs to bovine IVP systems improved embryo quality, which highlights the promising role of EVs as bioactive supplements for this field (225) Additionally, OF-EVs contain lipid mediators such as cholesterol and sphingomyelin that modulate membrane fluidity and support the acrosome reaction, further enhancing sperm-oocyte interaction (226).

### 8.4 Biomarkers of sperm fertility in EVs

The molecular biomarkers encapsulated within EVs present information that demonstrates both the level of sperm quality and fertility potential of affected males. For example, a differentiated pattern of the diagnostic potential expressing eight miRNAs exists between EVs released from normozoospermic and oligoasthenozoospermic men. Research demonstrates that prostasome-derived EVs contain Clusterin as a protein marker, which shows the ability to differentiate between fertile and subfertile males (178, 227). Moreover, RPL investigations of spermatic EVs from affected couples' partners showed 106 proteins decreased while 71 proteins increased, indicating impaired embryo development (228). In addition to Clusterin, other EV-associated proteins such as SPAM1, SEMG1, ANXA1, and HSP70 have been linked to sperm quality, motility, and fertilization capacity. Furthermore, tetraspanins (CD9, CD63), integrins (ITGA6), and enzymes like PDI and ENO1 have been studied as potential markers in seminal EVs, reflecting sperm functional status and fertility outcomes. Several small non-coding RNAs, including miR-30b, miR-151a, and piR-823, have also been identified in EVs from high- vs. low-fertility males (177, 229–231).

Sperm evaluation through EVs offers a method for assessment that shows potential for enhancing both diagnostic performance and therapeutic outcomes at IVF clinics. A summary table (Table 3) has been added to highlight key biomarkers, their roles, and associated literature references.

**Table 3:** Overview of extracellular vesicle (EV)-associated biomarkers linked to sperm fertility.

**No**.	**Biomolecule type**	**Biomarker name**	**Functional role**	**EV source**	**Reference(s)**
1	Protein	Clusterin	Differentiates fertile from subfertile males	Prostasomal EVs	(343)
2	Protein	SPAM1	Involved in sperm–egg binding	Seminal plasma EVs	(344)
3	Protein	SEMG1	Enhances sperm motility	Seminal plasma EVs	(345)
4	Protein	ANXA1	Stabilizes sperm membrane	Seminal plasma EVs	(346)
5	Protein	HSP70	Protects sperm from cryodamage and oxidative stress	Seminal plasma EVs	(347)
6	Protein	PDI	Involved in sperm function and protein folding	Sperm-derived EVs	(348)
7	Protein	ENO1	Supports energy metabolism in sperm	Sperm-derived EVs	(199)
8	Protein	CD9, CD63 (Tetraspanins)	Classical EV markers; involved in membrane fusion	Seminal and oviductal EVs	(177)
9	Protein	ITGA6 (Integrin α6)	Related to sperm adhesion and membrane fusion	Seminal plasma EVs	(349)
10	Protein	OVGP1	Modulates zona pellucida and promotes fertilization	Oviductal EVs	(207)
11	Protein	Gelsolin	Facilitates acrosome reaction and enhances motility	Seminal plasma EVs	(350)
12	Protein	PDIA4	Supports sperm-oocyte interaction and fertilization	Seminal plasma EVs	(231)
13	Protein	CRISP1	Improves motility and capacitation in asthenozoospermic men	Seminal plasma EVs	(171)
14	Protein	SNF8	Associated with poor semen quality	Seminal plasma EVs	(351)
15	Protein	Mucin 15	Linked to low fertility phenotype	Seminal plasma EVs	(352)
16	miRNA	miR-21-5p	Regulates capacitation and suppresses apoptosis	Seminal plasma EVs	(203)
17	miRNA	miR-222	Modulates apoptosis and sperm survival	Seminal plasma EVs	(353)
18	miRNA	miR-34c	Supports mitochondrial activity during cryopreservation	Oviductal and seminal EVs	(354)
19	miRNA	miR-19b	Reduces oxidative stress in cryopreserved sperm	Oviductal and seminal EVs	(355)
20	miRNA	miR-30b	Linked to high sperm quality	Seminal plasma EVs	(355)
21	miRNA	miR-151a	Correlates with sperm motility	Seminal plasma EVs	(356)
22	miRNA	Let-7 family (e.g., Let-7a, Let-7f)	Related to developmental competence and sperm integrity	Follicular and seminal EVs	(230)
23	miRNA	miR-10a, miR-29a	Potential diagnostic markers for male infertility	Seminal plasma EVs	(354)
24	piRNA	piR-823	Marker differentiating fertile vs. infertile men	Seminal plasma EVs	(357)

The table includes different types of biomolecules (proteins, miRNAs, piRNAs, and other small non-coding RNAs).

## 9 The role of extracellular vesicles in cumulus expansion

Cumulus expansion is a critical step in oocyte maturation and subsequent fertilization. Recent research has highlighted the involvement of EVs in mediating this process through the transfer of signaling molecules and genetic regulators. This section discusses the emerging role of EVs derived from various reproductive sources in modulating cumulus cell function, matrix remodeling, and oocyte developmental competence, with particular focus on their molecular mechanisms and applications in ARTs (232–234).

### 9.1 EVs enhance cumulus expansion and oocyte maturation

Multiple studies have shown that EVs isolated from FF FF-EVs, plasma, seminal plasma, and oviductal fluid influence the expansion of the cumulus cells through their effects on gene expression and cell division, as well as matrix remodeling. Notably, plasma-derived EVs have been shown to enhance both cumulus cell expansion and improve the oocyte maturation rate to the MII stage during *in vitro* maturation (22).

Similarly, exposure to bovine FF-EVs results in increased transcription of key cumulus expansion genes-PTGS2, PTX3, and TNFAIP6- in murine cumulus cells, suggesting a conserved mechanism across species (235). Moreover, the presence of proliferative and maturation-related molecules in plasma EVs activates HAS2 and PTGS2 expression, which speeds up cumulus expansion and oocyte developmental progression (22). These findings are consistent with evidence that FF-EVs enhance both the expansion of bovine cumulus cells and the maturation status of oocytes in IVM systems (235).

In addition, the expression profiles of bovine cumulus cells' miRNAs shift based on the progesterone levels and estrous cycle phase, which is mirrored by sEVs from FF (236). Of particular interest, sEVs from low-progesterone follicles demonstrate the ability to activate genes linked to reproduction and immune response, which indicates their value as biomarkers for determining oocyte competence (237).

Furthermore, seminal plasma EVs of both small and large subtypes interact with porcine cumulus cells, which affects their gene networks that control hormone production as well as cumulus expansion (238). Conversely, different studies have presented contradictory evidence about whether FF-EVs successfully stimulate the expansion of cumulus cells. While several studies have demonstrated positive actions regarding cumulus cell enhancement (239, 240). These discrepancies likely stem from species-specific differences in EV cargo composition, as well as variations in experimental design, EV isolation methods, and culture systems.

In equine models, it has been demonstrated that FF-EVs enhanced cumulus expansion through Method II, which used two-step IVM, but they did not benefit Method I, which depended on continuous cultures (90). Moreover, studies show that FF-EVs cause different impacts on compacted and expanded COCs; for instance, they enhance viability in compacted COCs while decreasing in expanded groups (241). These observations underscore the importance of timing and cellular context in determining the responsiveness of cumulus cells to EV signals.

Finally, the development of rabbit oocytes benefits from EVs derived from testis, prostate, and epididymis that induce an increase in KISS1, MDK, NTF3, ADAM17, and VEGFA factors, which support cumulus expansion and oocyte maturation (242).

### 9.2 Mechanisms of EV-mediated cumulus expansion

Extracellular vesicles enable cumulus expansion by transporting bioactive substances that direct extracellular matrix (ECM) formation and key signaling activities that support the developmental competence of the oocyte. The major mechanisms include: (1) firstly, the activation of expansion-related genes constitutes the first mechanism through which EVs affect ECM development by stimulating the expression of HAS2 and PTGS2 genes along with PTX3 and TNFAIP6 factors (243). (2) Secondly, the mitogen-activated protein kinase (MAPK) pathway is regulated through EV-delivered proteins and miRNAs that activate this pathway to initiate cumulus expansion, together with cell differentiation processes (89). (3) Thirdly, the WNT signaling regulatory mechanism involves WNT signaling pathway control by EVs released from follicular and oviductal origins that facilitate follicular development and control cumulus cell functions (175). (4) Lastly, the small vesicles deliver miRNAs to target cumulus cells to modify their genetic expression, which affects their biological activities along with oocyte developmental readiness. The miRNA expression patterns in cumulus cells differ based on the maturation phase of the releasing follicle, according to research (236).

Collectively, FF-EVs demonstrate great potential for ART applications through their function as minimally invasive markers that help measure oocyte quality and developmental potential. When applied appropriately, adding appropriate doses of FF-EVs and plasma-derived EVs to IVM media enhances both cumulus cell homeostasis and the oocyte maturation process, which leads to superior blastocyst production in IVP systems, according to the literature (243, 244). However, the wide range of EVs impacts between species requires researchers to establish separate optimal EVs utilization procedures for each species that aims to use them in IVM and IVP research. Therefore, to maximize the beneficial effects of EVs and outcomes from reproductive technology, it requires a dedicated assessment of the EVs' source type together with their concentration levels, developmental stage of application, and target cell condition.

## 10 The role of EVs in granulosa cell proliferation

Oocytes require the specialized somatic cells known as granulosa cells (GCs), which surround them and form the follicular structure together with theca and cumulus cells. GCs are vital for oocyte growth and follicular development, and steroid hormone production. Specifically, the oocyte resides within these granulosa cells, which develop into single or multiple layers within the follicular wall that support follicular growth as well as produce estrogen and progesterone, and establish oocyte competence (245, 246).

Importantly, follicular expansion and oocyte development depend on the proliferation of granulosa cells, a process regulated by paracrine factors as well as EVs regulate (247).

Recent studies have shown that EVs function as vital mediators supporting granulosa cells proliferation and follicular function. For instance, it has been revealed that EVs separated from FF promoted granulosa cells proliferation at different rates, depending on the follicular origin and size of the antral follicles (68, 245). Moreover, the bioactive cargo of EVs function as key regulators for cellular functions necessary for follicular development. The received molecules modify cell cycle regulatory mechanisms as well as differentiation processes and metabolic functions after entering recipient cells (190, 248).

Specifically, studies have identified miRNAs (e.g., miR-21, miR-26a), proteins such as GDF9, BMP15, and metabolic enzymes like ENO1 within FF-derived EVs, which enhance GC proliferation and function (249).

miR-21, for example, has been shown to promote GC survival by suppressing pro-apoptotic genes, while GDF9 and BMP15 are known to activate SMAD signaling in granulosa cells (203).

In terms of uptake, granulosa cells internalize EVs by means of two dominant endocytic pathways known as clathrin-mediated endocytosis and caveolae-mediated endocytosis. Together, paracrine signals from the oocyte strengthen the vesicle entry process by working with membrane invaginations through the combined action of clathrin-coated pits and plasma membrane caveolae (250–252).

Additionally, other internalization mechanisms like macropinocytosis and phagocytosis contribute to EV uptake pathways which influence subsequent functions of the granulosa cells (253, 254). Once internalized, EVs cargo activates intercellular signaling pathways that drive granulosa cells proliferation and activity.

Notably, the activation of MAPK pathway by EVs frequently occurs as it significantly influences granulosa cells proliferation together with differentiation. Similarly, studies indicate that granulosa cells proliferation from EV signaling likely requires additional signaling networks like Src kinase and phosphoinositide 3-kinase (PI3K) yet scientists need to fully clarify their precise effects (253–255).

FF-EVs have been shown to influence the expression of FSH receptor and aromatase (CYP19A1) genes, thereby modulating the endocrine function of granulosa cells (256).

Furthermore, it has been shown that EVs affect granulosa cell proliferation patterns and this affects the outcomes of both IVEP and ARTs. Through their actions, microvesicles perform dual actions that first stimulate follicular development before they enhance hormonal communication which leads to better quality oocytes and higher maturation rates and embryonic development during *in vitro*.

Consequently, studies indicate that EVs demonstrate promising indications as fertility markers and create new possibilities to enhance IVM protocols across various species. However, it remains essential to determine the particular molecular elements inside EVs that promote granulosa cells proliferation together with how these compounds impact follicles differently between diverse animal models.

## 11 The role of EVs in oocyte maturation

The process of oocyte maturation involves crucial female reproductive development because it makes an immature oocyte prepared for fertilization. This maturation process occurs within the ovarian follicle, where the maturation process develops and remains under hormonal control and intercellular signals with specific molecular pathways doing the regulation (257–259). Following meiotic resumption, the oocyte moves from germinal vesicle stage to MII stage while chromosomal condenses and the first polar body extrusion while experiencing major cytoplasmic changes. Throughout this process, the oocyte competence becomes possible due to the combination of metabolic support and paracrine signaling from granulosa and cumulus cells (260–262).

During *in vitro* maturation the resumption of meiosis starts prior to complete cytoplasmic maturation potentially leading to diminished developmental competence. Notably, the success of fertilization and the subsequent embryo development depends on three critical aspects of cytoplasmic maturation: mRNA accumulation and organelle redistribution, and metabolic adjustments. The second messenger, cyclic adenosine monophosphate (cAMP) functions as the fundamental regulator which sustains meiotic arrest in the follicle. However, the reduction in cAMP after follicular removal leads to meiotic resumption. Therefore, to improve IVM results, it is crucial to postpone nuclear maturation while completing cytoplasmic maturation by the modification of cAMP levels (263–266).

Recent research has demonstrated that EVs act as vital messengers between follicles to transfer microenvironment signals that control oocyte development. The miRNAs together with proteins and lipids from FF, oviductal secretions, and cumulus cells act as controlling factors during oocyte maturation. Research indicates that EVs have distinctive effects on oocyte maturation across different species (267). For example, the interaction of EVs derived from seminal plasma with porcine cumulus cells during IVM shows no significant impact on oocyte maturation rates. Interestingly, it has been identified that a particular group of EVs referred to as large EVs that affect steroidogenesis-related genes which influence the functionality of cumulus cells (238). Conversely, mice plasma-derived EVs show the ability to accelerate oocyte maturation while indicating potential improvements for developmental competence (22).

In bovine studies, supplementation of FF-derived EVs during IVM has been shown to significantly increase blastocyst yield and oocyte maturation rate. Equine FF-EVs have also enhanced COC viability in compacted follicles, indicating species-specific functional responses. Furthermore, supplementation with oviductal EVs improved cytoplasmic maturation markers including mitochondrial redistribution and cortical granule alignment in bovine oocytes (180, 268).

Moreover, MAPK signaling pathway acts as a principal pathway for EV-mediated oocyte maturation and cumulus expansion (269, 270). In particular, EVs that carry particular miRNAs, including miR-21, miR-378, and miR-146a, which efficiently regulate MAPK activity, induce oocyte maturation and improved cumulus cell function. Similarly, evidence shows EVs affect the PI3K/AKT signaling cascade which enables metabolic coupling and maintains mitochondrial function between oocytes and cumulus cells (271–273).

Other candidate molecules include TGF-β1, BMP15, and GDF9-related transcripts delivered via FF-EVs, which are linked to cumulus expansion and oocyte competence.

In addition to direct contact, oocyte–granulosa cell communication is mediated by paracrine signaling and EV-mediated pathways. This dual mode of communication through gap junctions and transzonal projections enables precise control of maturation processes (274, 275). It has been shown that the molecular contents of EVs, establish the direction of developmental progression for oocytes and cumulus cells and administer their cellular functions (276, 277). Remarkably, recent investigations confirm EVs with miRNAs can enter oocytes by penetrating the zona pellucida without transfection agents so they may offer clinical value to IVM protocols (278–280).

Given these findings, EVs have a significant capacity to regulate oocyte maturity, which would increase the success rates of IVEP and ART treatments. By synchronizing the nuclear and cytoplasmic maturation, EVs promote improved development competence while enhancing fertilization results and blastocyst development. Consequently, the utility of EV-based strategies creates biologically accurate protections for *in vivo* follicles which enhance the performance of IVM systems across different animal species.

## 12 The role of EVs in embryo development and quality

In both natural conception and ARTs, embryo development and quality are critical factors that determine successful implantation and pregnancy outcomes. Recent research has emphasized EVs as important regulators of early embryonic development which they function as intercellular messengers that alter gene expression and cellular communication.

### 12.1 EVs in preimplantation embryo development

During early embryogenesis, embryos produce a milieu containing biochemical molecules, including EVs, which facilitate both autocrine and paracrine functions. In the porcine model, it has been shownthat developing embryos release exosomal marker CD9 along with vesicles measuring 30–120 nm in diameter, consistent with the size of exosome. These vesicles deliver mRNAs, including OCT4, SOX2, and KLF4 at different developmental stages of embryonic development. Moreover, embryonic vesicles have the capability to cross the zona pellucida before being taken up by blastomeres, supporting intercellular communication during embryonic development (76, 197, 276). Nevertheless, scientists still need to identify all the mechanisms through which embryo development depends on EVs.

Recent studies have demonstrated that supplementing embryo culture media with EVs derived from maternal reproductive tract fluids—especially oviductal and uterine EVs—can significantly enhance blastocyst formation, hatching rates, and overall embryo quality. For example, bovine oviductal EVs added during early cleavage stages improved blastocyst rates and upregulated key pluripotency markers such as OCT4 and NANOG (281). Similarly, in porcine embryos, oviductal EVs enhanced mitochondrial activity and cell proliferation, supporting superior blastocyst development. Uterine EVs collected during the peri-implantation phase have also been shown to promote trophoblast elongation and differentiation by delivering integrins, growth factors, and miRNAs. These findings highlight the importance of EV source and developmental timing when applying EVs to *in vitro* production (IVP) systems (187, 282).

Similarly, EVs in reproductive fluids such as FF and AOF transport miRNAs, proteins together with lipids which contribute to embryo development. A study by Asaadi et al. (16) showed that EVs obtained from both FF and AOF fluid improved blastocyst quality by enhancing TE and ICM development as well as reducing the apoptotic cell ratio. In addition, studies have shown that in the bovine model, epithelial EVs derived from the oviductal tissue improve embryo quality and increase total cell numbers, and enhance vitrification survival rates (79, 283).

Furthermore, embryonic gene expression is fundamentally modulated by the miRNA content carried by EVs. For instance, investigations have identified two particular miRNAs named miR-21 and miR-2861 which demonstrate potential in enhancing embryo quality alongside development. Conversely, research indicates that embryonic development can be negatively affected by EVs contents, such as miRNA-146b, which has been shown to impair embryo development (284–286).

In addition to maternal sources, embryo-derived EVs also appear to act in an autocrine manner to modulate their own development. For instance, EVs secreted by preimplantation embryos have been shown to influence cell lineage allocation, possibly by redistributing miRNAs and lncRNAs that regulate transcriptional activity in blastomeres. Moreover, beyond miR-21 and miR-2861, other miRNAs such as miR-320a and miR-30c have been associated with improved blastocyst viability and reduced apoptosis in both mouse and bovine models (287–289).

On the contrary, studies have found that excessive expression of miR-155 or miR-146b in EVs is associated with reduced developmental rates and impaired ICM formation. These insights suggest that EV content profiling may help screen for supportive vs. detrimental signals in embryo culture (285).

### 12.2 EV-mediated communication between the embryo and the oviduct

Effective communication between the embryo and the oviduct is crucial for proper development. The developing embryo requires optimal environmental conditions provided by, the oviduct, while its secretions-particularly EVs-function as essential mediators in the interaction between embryo and oviduct (77, 290). These EVs carry specific molecular cargos including proteins (e.g., oviductin), lipids, and miRNAs (e.g., miR-30c, miR-375), which modulate gene expression and support embryo development (74). Moreover, embryo physiology influenced by vesicles isolated from various region of the oviduct, such as ampulla and isthmus. For example, EVs from the ampulla region promote early cleavage, while isthmus-derived EVs enhance blastocyst formation and quality in bovine and porcine models (287). Oviductal EVs modify their miRNA miRNA composition in response to the presence of an embryo, indicating bidirectional communication between embryo and oviduct (291–293). Transcriptomic analysis has revealed altered miRNA profiles in oviductal EVs, such as increased let-7a and miR-200 family members, reflecting maternal adaptation to embryonic signals (294).

Similarly, the culture media of developing bovine preimplantation embryos contains EVs, whose size and concentration are associated with embryonic quality. Studies have shown that higher concentrations of embryo-derived EVs (30–150 nm) are correlated with improved ICM/TE ratio and mitochondrial activity (207). Therefore, it has been proposed that these EVs could be used to assess embryonic competence along with predicting implantation outcomes using these EVs (295–297).

## 13 The role of EVs in embryo hatching and pre-implantation development

**Figure 5:**
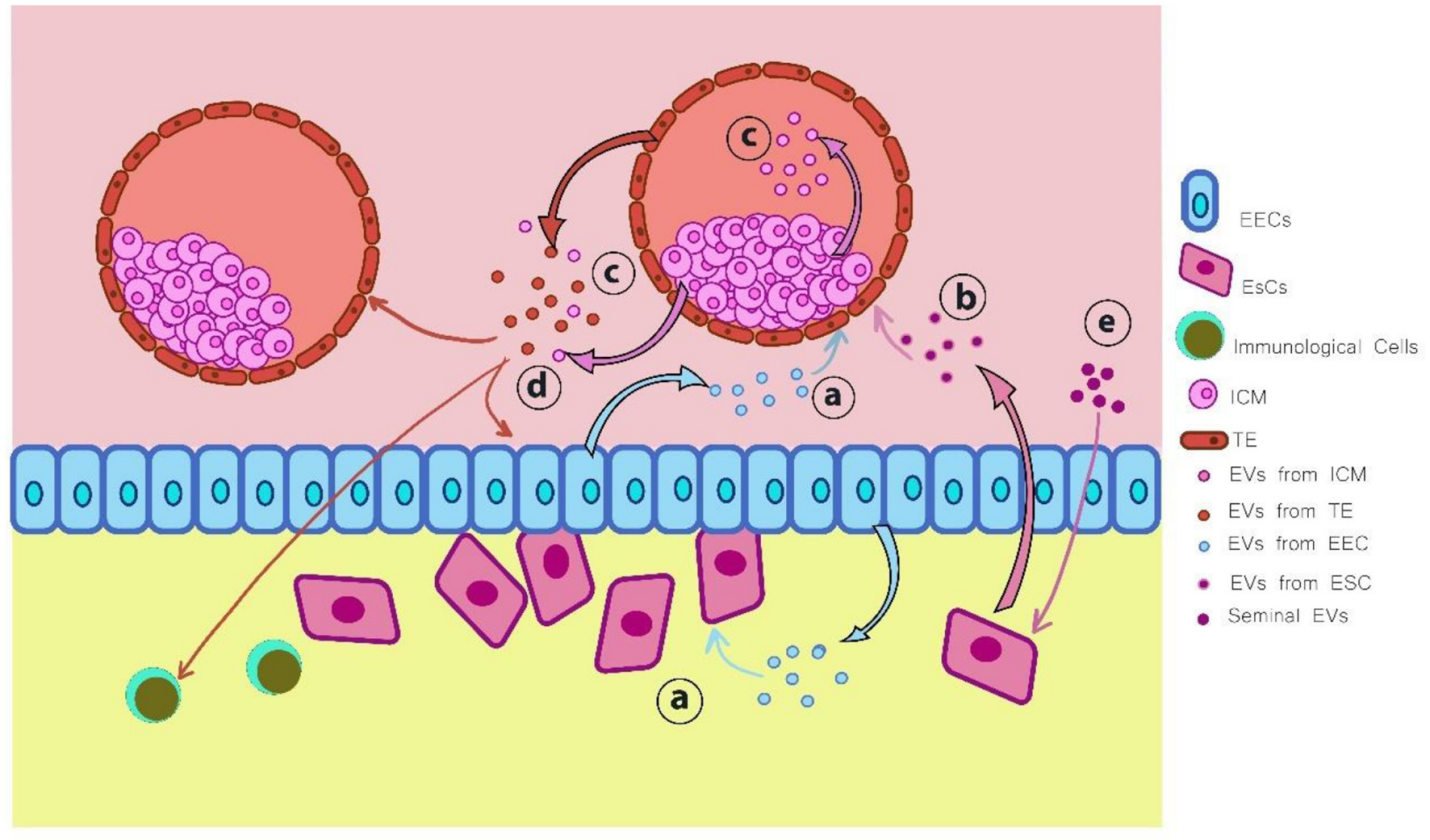
Extracellular vesicles (EVs) are secreted by various types of cells during the implantation process: **(a)** EVs derived from endometrial epithelial cells (EECs) interact with both the embryo and uterine fibroblasts; **(b)** EVs secreted by endometrial stromal cells (ESCs) influence embryonic development; **(c)** EVs originating from the inner cell mass (ICM) act on the trophectoderm (TE) and can also be transferred outside the blastocyst; **(d)** EVs produced by the embryo, including those from both TE and ICM, modulate the function of EECs, neighboring embryos, and immune cells; **(e)** Seminal EVs exert regulatory effects on ESCs. This figure was inspired by the work of Chen et al. (211). Illustration created using Digital Paint and Adobe Photoshop 2023.

Embryos must undergo hatching to exit their zona pellucida envelope during pre-implantation development (Figure 5). A successful hatching process not only allows embryonic development to continue but also develops implantation competence. One of the main challenges during IVEP involves establishing optimized conditions that promote embryo hatching and developmental success (358, 359).

Interestingly, both embryos and the maternal reproductive tract secrete EVs, which have been shown to regulate early developmental processes. Assessment data show that pre-implantation embryos actively release embryo-derived EVs into the *in vitro* culture medium throughout their development (360, 361). For example, Giacomini et al. (86) proved that pre-implantation human embryos produce EVs that express CD63, CD9, and ALIX markers which carry stemness-related gene transcripts and HLA-G protein. The identified embryonic EVs show potential as self-regulatory agents that may help blastocysts communicate with their neighbors before implantation occurs.

Furthermore, research using bovine IVEP models has shown that embryonic vesicles promote both blastocyst development and the hatching process. Specifically, miRNA-378a-3p, present within embryonic EVs, plays a regulatory role in bovine blastocyst hatching (298). In addition, recent studies suggest that circular RNAs also play a developmental role in embryos. For instance, circAGO2 is proposed to function as a binding molecule for RNAs involved in EVs-mediated communication, which may influence blastocyst hatching (86). Moreover, it has been reported that bovine embryo hatching rates improved following the inhibition of the small RNA tsRNA tDR-14:32-Glu-CTC-, which was also associated with altered gene expression revealed to hatching (78). Consequently, the cargo of embryonic vesicle cargoes contribute to embryonic developmental competence and holds promise for improving embryo quality IVEP systems.

EV-mediated signaling not only guides embryonic development through embryo-derived vesicles but also reflects inputs from maternal reproductive conditions. Interestingly, studies have revealed that endometrial EVs are present in uterine fluid across menstrual and estrous cycles, with their concentration peaking during the implantation window (299–303).

Proteomic analysis further show that the protein content of endometrial EVs varies according to hormonal fluctuations throughout the menstrual cycle (299). Furthermore, epithelial-origin endometrial cells have been shown to absorb EVs from recipient cells, resulting in receptor-modulating effects (304). Additionally, studies have found that EVs are enriched with proteins associated with extracellular matrix remodeling, cell adhesion, and immune modulation, all of which are essential for embryo-endometrium interactions *in vivo* (305–312). As a result, researchers are exploring the utilization of endometrial EVs in IVEP systems, which the aim of enhancing embryo competence and improving implantation outcomes.

In the same context, EVs isolated from ruminant reproductive tracts are of particular scientific interest due to their role in supporting pre-implantation embryos. In sheep, conceptus survival and pregnancy recognition are influenced by EVs present in uterine luminal fluid, which also regulate modulate immune responses (313). Likewise, bovine uterine EVs appear to affect trophoblast-endometrial communication by delivering conceptus-derived interferon tau, which promotesendometrial receptivity (314). Incorporating embryonic vesicles into embryo culture systems helps creates an environment that more closely mimics physiological conditions, thereby minimizing negative effects from laboratory culture on embryonic development (315).

Applications of exosomes in ARTs show promising translational potential by improving both embryo assessment and culture environments. Currently, embryo grading depends primarily on morphological criteria and invasive biopsy methods, which often fail to accurately identify embryos with high implantation potential (316, 317). Conversely, EVs profiles differ between viable and degenerate embryos, suggesting that EVs may serve as non-invasive biomarkers for assessing embryo competence (15). Moreover, the assessment of embryonic development in IVEP now incorporates EVs derived from stem cells and reproductive tract secretions (318).

Despite being in early stages, available evidence strongly supports the vital importance of EVs in embryonic development despite early stages of functional research in IVEP. Future research should focus on optimizing EV-based techniques to improve IVEP outcomes-including enhanced culture conditions, better embryo selection methods, and novel therapeutic applications. Obviously, the development of improved EVs isolation and characterization methods will open new avenues for their integration into ART.

## 14 Conclusion

Extracellular vesicles are increasingly recognized as critical modulators in the context of *in vitro* embryo production, facilitating vital intercellular communication throughout the processes of oocyte maturation, sperm functionality, fertilization, and embryonic development. By carrying bioactive molecules, including proteins, microRNAs, and lipids, EVs exert an impact on gene expression and cellular dynamics in both gametes and embryos. They serve not only as messengers but also as promising tools for diagnosis and therapy in assisted reproductive technologies.

Moreover, EVs hold promise as non-invasive biomarkers for assessing gamete and embryo quality, offering safer and more precise alternatives to current invasive methods.

Notwithstanding their considerable potential, obstacles persist concerning the standardization of EV isolation, characterization, and application across diverse species and clinical environments. Further investigation is needed to understand how EV heterogeneity across different follicular stages and species influences reproducibility and functional outcomes. Advancing analytical technologies such as single-vesicle profiling, multi-omics integration, and real-time EV tracking will be critical in unlocking their full potential.

Future studies should focus on revealing the specific mechanisms underlying EV functionality and enhancing their integration into IVEP contexts. Eventually, the incorporation of EV-based methodologies has the potential to enhance embryo quality, elevate implantation success rates, and contribute to improved reproductive outcomes in both agricultural and human fertility interventions. Ultimately, the development of EV-based supplements or engineered culture systems could significantly improve embryo viability, implantation success rates, and long-term ART outcomes.
